# Structural model of silicene-like nanoribbons on a Pb-reconstructed Si(111) surface

**DOI:** 10.3762/bjnano.8.185

**Published:** 2017-09-05

**Authors:** Agnieszka Stępniak-Dybala, Mariusz Krawiec

**Affiliations:** 1 Institute of Physics, M. Curie-Skłodowska University, Pl. M. Curie-Skłodowskiej 1, 20-031 Lublin, Poland

**Keywords:** density functional theory (DFT), scanning tunneling microscopy (STM), silicene, Si nanoribbons

## Abstract

A structural model of the recently observed silicene-like nanoribbons on a Pb-induced √3 × √3 reconstructed Si(111) surface is proposed. The model, which is based on first principles density functional theory calculations, features a deformed honeycomb structure directly bonded to the Si(111) surface underneath. Pb atoms stabilize the nanoribbons, as they passivate the uncovered substrate, thus lower the surface energy, and suppress the nanoribbon–substrate interaction. The proposed structural model reproduces well all the experimental findings.

## Introduction

The discovery of the exotic nature of graphene [1–2] has stimulated a growing interest in similar materials with a two-dimensional (2D) honeycomb geometry, mainly composed of group-IV elements [3–6]. In particular silicene, a silicon counterpart of graphene, has attracted increasing attention due to its compatibility with existing semiconductor technology [7–12].

After the theoretical predictions [13–15], a great number of experimental studies has been devoted to the fabrication of silicene, but still the synthesis of this material remains a big challenge. So far a freestanding layer has not been produced. However, mainly epitaxial layers have been synthesized on Ag(111), Ir(111), ZrB_2_(111) [16–24] or recently on graphite [25]. Among them epitaxial silicene on Ag(111) has been the most extensively studied. Depending on the temperature and deposition rate, various superstructures, i.e., 4 × 4, 
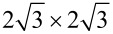
 and 
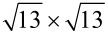
 (with respect to the Ag(111) lattice) have been observed [19,26]. The 2D honeycomb structure of silicene layer with 4 × 4 symmetry on Ag(111) is well established and supported by many experimental and theoretical results [16–19,27–31]. Other phases of Si/Ag(111) are more controversially discussed, while the problem of the silicene formation on other substrates has been addressed only in a few reports. Nevertheless, the first silicene-based field effect transistor device operating at room temperature has already been demonstrated [12].

To get a deeper and more detailed insight into the physics and chemistry of silicene/substrate systems density functional theory (DFT) calculations have usually been required. In all these cases silicene was reported to be formed in 2D domains with a corrugated hexagonal structure. Nevertheless, not all experiments, most notably on Ag(111), support the scenario of silicene formation, mainly due to a problem with electronic properties. Therefore there is still a significant amount of skepticism about this issue. In many cases density functional theory (DFT) calculations were required to get more detailed information on what structures have really been obtained. The same problem concerns Si nanoribbons (NRs) grown on the Ag(110) surface [32–40]. The scanning tunneling microscopy (STM) images show isolated 1.6 nm wide ribbons [32,35,41]. However, no hexagonal structure is visible in the STM topography. First DFT calculations proposed a hexagonal structure of the Si NRs. However, recent theoretical and experimental studies [42–44] have found this structure to be incorrect, and opt for the so-called “pentamer” model, in which Si atoms are arranged into chains composed of pentagonal rings running along the rows. Thus, in most cases the structure of the deposited silicon is governed by the underlying substrate.

Alternative substrates that could host silicene without destroying its remarkable electronic properties are still highly required. Recently, we have made attempts to grow silicene on a Pb substrate because the results of the DFT calculations of [45–46] were very promising in view of silicene formation. We started from the thinnest Pb substrates, which are 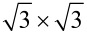 and 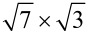 reconstructions of Pb on Si(111). Our STM studies on the Pb-reconstructed Si(111) surface revealed that deposited Si atoms form wide nanoribbons [47]. The NRs, running in three high-symmetry directions of the Si(111) surface, are several nanometers long, 1.6 nm wide and show a local 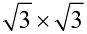 reconstruction. Although no details on the atomic structure existed, these nanostructures have been interpreted in terms of silicene-like nanoribbons grown on the bare Si(111) surface [47].

In the present work we focus on the determination of the atomic structure of the 1D Si NRs grown on the Pb-induced 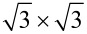 reconstructed Si(111) surface, in short 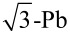. Our combined STM and DFT studies confirm the proposed scenario of silicene-like NRs. In particular, the DFT calculations reveal the hexagonal structure of the Si nanoribbons, which are directly bonded to the bare Si(111) surface. However, Pb atoms play an important role in stabilizing the structure, as they lower the surface energy. The proposed structural model features a deformed honeycomb structure in reversed AB registry with respect to the top Si(111) substrate layer, and reproduces well the experimental data. These findings provide a deeper insight into the formation of silicene nanostructures on metal-stabilized silicon surfaces, and may serve as help for the growth of silicene on other substrates.

## Experimental and Computational Details

All the measurements have been done under UHV conditions with a ^4^He-cooled scanning tunneling microscope (Omicron) working at 4.5 K. For STM/STS measurements electrochemically etched tungsten was used. The Pb/Si(111) sample was prepared in situ by evaporation of Pb on the Si(111)-7 × 7 substrate. Next, the Si layer was deposited onto the sample held at 200 K within 20 min. Details of the preparation can be found in [47]. The presented nanoribbons were obtained by two-step annealing: first at room temperature for 1 h, and then by direct heating at around 400 K for 5 min.

Density functional theory calculations were performed within the Perdew–Burke–Ernzerhof (PBE) [48] generalized gradient approximation (GGA) using projector-augmented-wave potentials, as implemented in the VASP (Vienna ab initio simulation package) [49–50]. The plane wave energy cutoff for all calculations was set to 340 eV, and the Brillouin zone was sampled by a 5 × 2 × 1 Monkhorst–Pack *k*-points grid [51], 640 eV and 9 × 3 × 1 grid in convergence tests, which resulted in changes of the surface energies of less than 0.1 meV/Å^2^. The spin–orbit interaction has not been included in calculations.

The Si(111) system has been modeled by eight Si double layers. To avoid the interaction between neighboring Si NRs, a 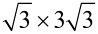 unit cell was used in calculations. Si atoms in the bottom layer were fixed at their bulk ideal positions and H atoms were used to saturate Si dangling bonds of the bottom layer, maintaining correct Si–Si bonds, to mimic bulk Si crystal. The positions of the remaining atoms were fully relaxed until the largest force in any direction was below 0.01 eV/Å. All the calculations have been performed in the same unit cell with fixed bulk Si lattice constant.

Based on the obtained electronic structure data of the Si NRs/Si(111) system described above, scanning tunneling microscopy simulations were performed by using the Tersoff–Hamann approach [52].

## Results and Discussion

**Figure 1:**
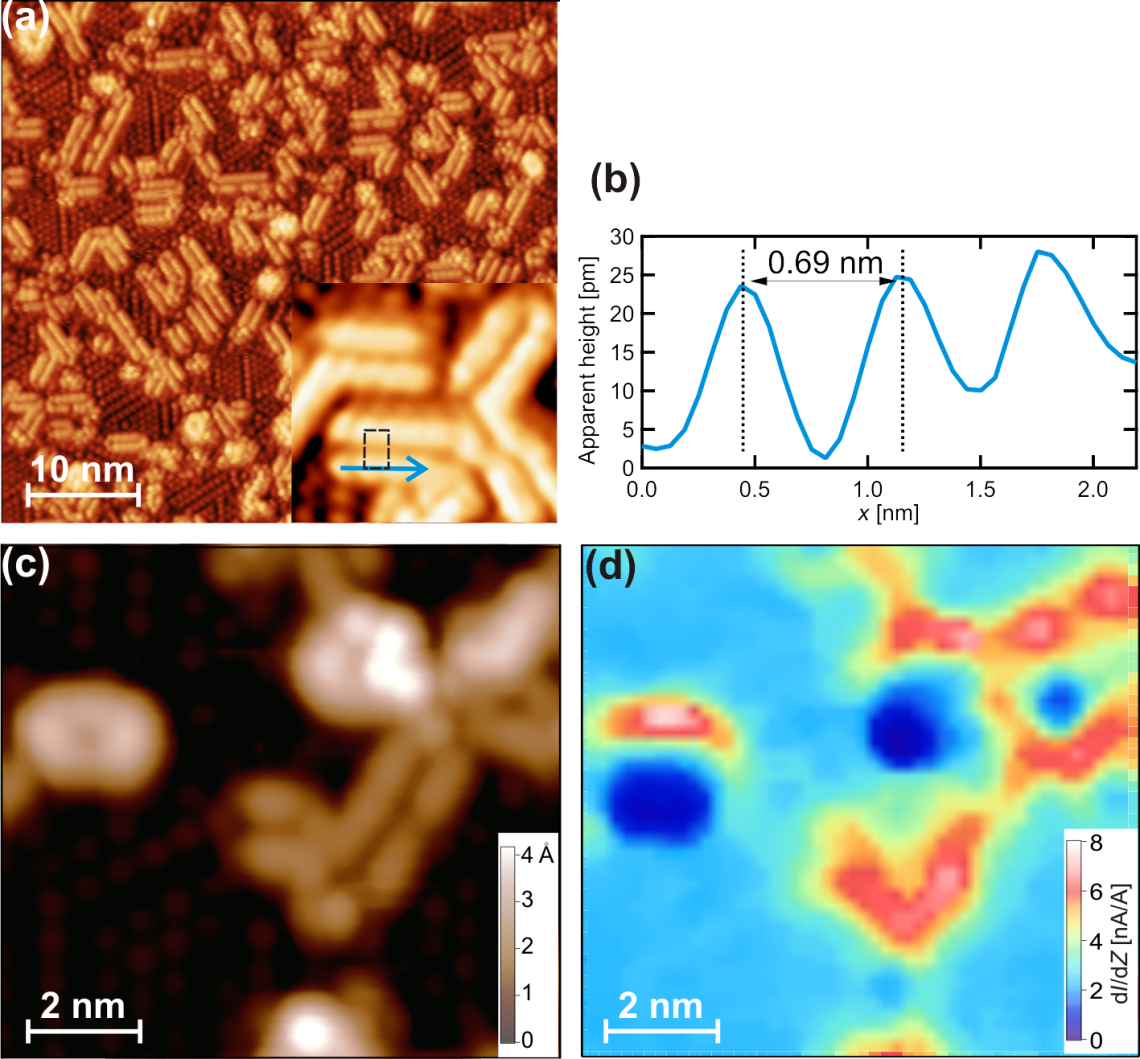
(a) STM topography (*U* = 2.0 V, *I* = 0.5 nA) of Si nanoribbons on a Si(111)
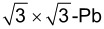
 surface. (b) Line profile along blue arrow marked in the inset of (a). The arrow points in the 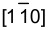 direction. The unit cell of a nanoribbon is also marked in the inset of (a). (c, d) Results of a simultaneously measured topography and d*I*/d*z* map of the same area. Scanning parameters were *U* = 1 V, *I* = 0.5 nA.

Typical Si nanoribbons are several nanometers long and run in one of three high-symmetry directions of the Si(111) surface. Figure 1a shows an example of such NRs as revealed by STM topography measurements. The NRs consist of Si atoms directly adsorbed on the Si(111) surface, as it was argued in [47], based on geometry considerations and STM measurements. It is also known, that Pb atoms strongly diffuse on Si substrates [53–54], so they can easily make room for growing Si NRs. Furthermore, different STS characteristics acquired on top of the NRs and in between them also point against Pb-composed nanoribbons. The experimental findings suggest that the observed nanostructures are wide nanoribbons rather then separated Si chains, as the inter-chain separations of 0.86 Å cannot be assigned to any Si–Si distance, and the modulations of STM topography across and along the nanostructures are very similar to each other. Moreover it is difficult to explain why the chains always grow in pairs.

An additional argument for the Si nature of nanoribbons can be provided by measurements of the local work function (Φ). In a first approximation Φ is proportional to the derivative of the tunneling current (*I*) with respect to the STM tip–sample distance (*z*) [55]. Thus, changes of Φ should be reflected in recorded d*I*/d*z* maps. Figure 1c,d shows topography and d*I*/d*z* maps simultaneously measured in the same area of the sample. A clear correlation between these quantities is observed. It is evident that the nanostructures feature a higher work function than the 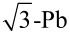 substrate, which suggests the NRs are composed of Si atoms. However, one has to remember that different values of Φ do not necessarily mean different chemical compositions. Thus the changes of the d*I*/d*z* values alone should be considered as necessary rather than sufficient condition. Nevertheless, the assumption of Si NRs is also in line with the Φ ordering of Pb and Si crystals, and with the calculated values of Φ, as it will be discussed later.

The internal structure of each NR, as revealed by STM topography measurements, consists of bright protrusions (BPs) periodically arranged within a NR. The BPs form a 6.9 Å × 8.6 Å rectangular lattice, marked in Figure 1a. Occasional zig-zag patterns have also been observed, but only in the presence of defects. The 6.9 Å periodicity matches well the length of the Si(111) 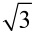 unit cell, which yields 6. Å, thus it can be assigned to the 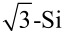 reconstruction. This reconstruction of Si can be obtained while growing Si structures directly on a Si(111) surface [56–57]. The reconstruction is also known to be realized in the case of multilayer silicene [58]. Its characteristic feature is an almost flat Si layer with sticking out Si atoms. These atoms give rise to a strong STM signal and are visible as BPs in topography images. They should not be misinterpreted as adatoms, since being shifted vertically they, in fact, still occupy honeycomb lattice sites. Such arrangement of atoms reflects a natural tendency of Si towards sp^3^-bonding [45,59].

Associating distance between BPs across NRs is a more complicated issue. The 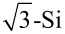 periodicity is achieved along the armchair (AC) direction, and BPs form a rectangular lattice, thus the distance of 8.6 Å must be associated with sticking out Si atoms along the zig-zag (ZZ) direction. However, the value of 8.6 Å does not fit any Si–Si distance on the Si(111) surface. In fact it is by 0.9 Å longer than the double lattice constant in the 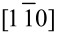 direction. Thus, likely the NRs structure will consist of deformed hexagonal rings. It could also be possible that the atomic structure includes pentagons, as in the case of Si NRs on the Ag(110) surface [42–44]. However, such scenario is less favorable for symmetry reasons since NRs grow directly on the Si(111) surface and the bonding of Si atoms arranged in pentagons to those in hexagons is expected to be energetically unfavorable.

Having collected information on details of NRs from experiments, we are ready to construct a structural model. First we neglect presence of Pb atoms and focus only on Si NRs grown on the bare substrate. As we already discussed, the nanoribbons are Pb-free objects, while Pb atoms themselves appear to be important only in the process of growing Si NRs and prevent Si structures from growing in a 3D fashion. The role of Pb will be discussed later.

We have considered a number of initial atomic structures of NRs composed of hexagons, pentagons or both building blocks. In the following, the lowest-energy structural models are labeled according to the number of hexagonal and pentagonal rings per unit cell forming a NR. The relative surface energies γ_NR(Si)_ and distances between BPs *d*_BP_, if available, of some representative structural models are listed in Table 1.

**Table 1:** Relative surface energies γ_NR(Si)_ and BP–BP distance *d*_BP_ of structural models of Si NRs on the Si(111) surface. γ_NR(Si)_, defined by Equation 1, is measured with respect to the energy of the 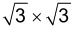 reconstructed bare Si(111), set as the energy zero. The models are labeled according to the number of hexagonal and pentagonal rings per unit cell constituting a NR. In some models only a single BP in the unit cell appears or there are no BPs at all. Then *d*_BP_ could not be determined.

model	γ_NR(Si)_ (meV/Å^2^)	*d*_BP_ (Å)

3hex	5.74	3.97
1hex-2pent	8.49	—
4hex	4.92	—
5hex	4.02	8.00
4hex-1pent	8.58	—

The surface energy γ_NR(Si)_ is defined as

[1]
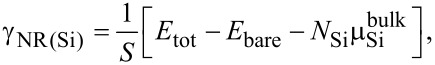


where *E*_tot_ and *E*_bare_ are total energies of the NR on the Si(111) surface and on the Si(111) surface with 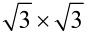 reconstruction. *N*_Si_ stands for the number of Si atoms in a NR, while 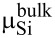 is the chemical potential of a Si atom, taken as its bulk value. The area of the surface unit cell is denoted as *S*.

All the structures listed in Table 1 feature positive values of γ_NR(Si)_, which means that they are less stable than the bare 
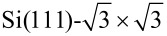
 surface. This suggests that the presence of Pb might be important in stabilizing NRs. Furthermore, it is clear, that NRs containing pentagonal rings are not preferred, as pentagons substantially increase the surface energy. This result confirms our expectation that pentagons do not fit well to the hexagonal structure of the Si(111) surface and that pentagonal objects should be less favorable. Another argument against pentamer-structure models is the lack of sticking out atoms in obtained structures. Thus, none of these models will reproduce the STM topography.

**Figure 2:**
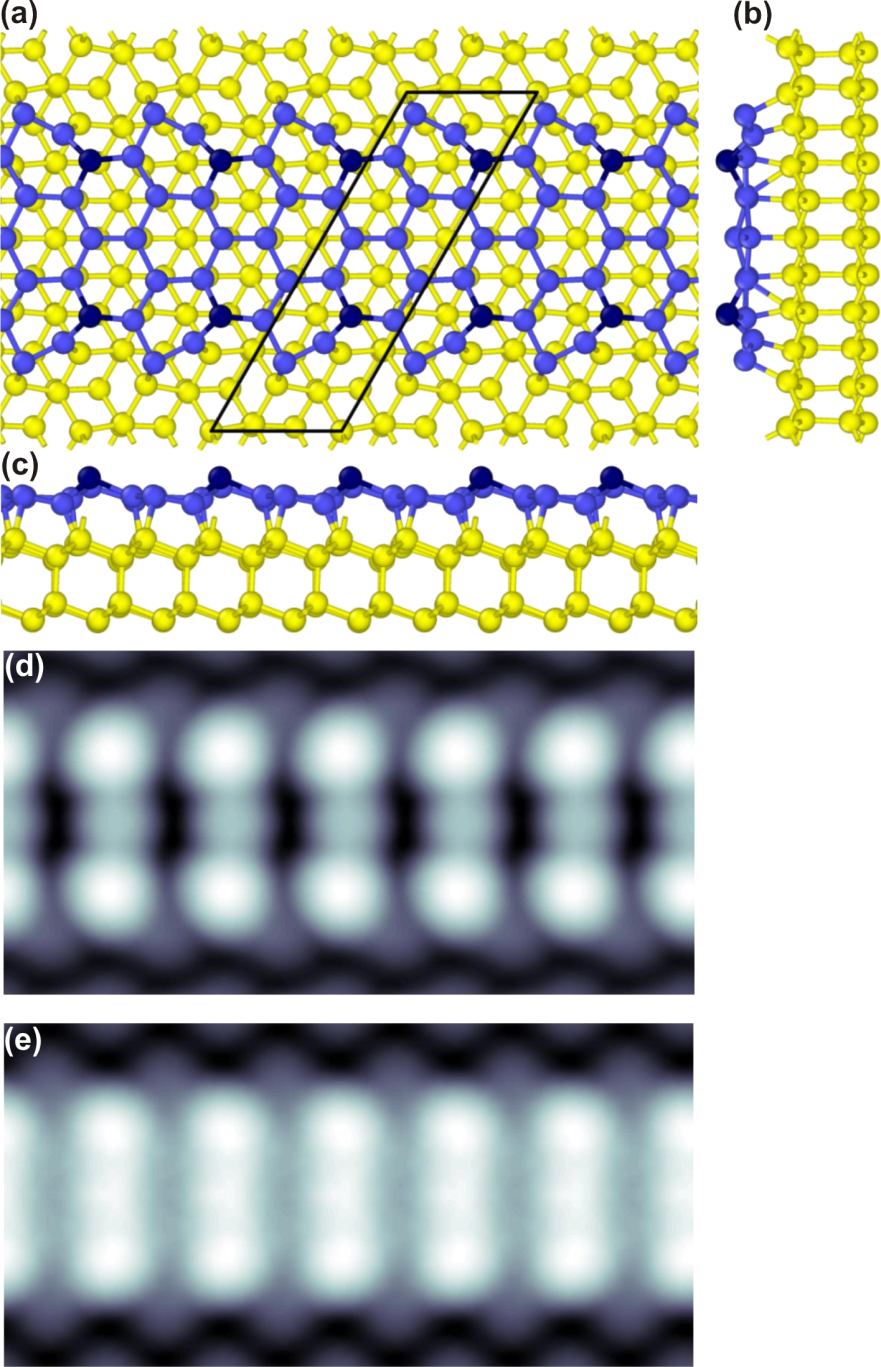
(a) Top and (b), (c) side views of the structural model with the lowest energy (5hex) of Si nanoribbons on a Si(111) surface. Different colors represent Si atoms of different parts of the structure: Atoms shown in blue constitute the Si NR, with sticking out atoms colored in dark blue, while yellow atoms represent the substrate. The black parallelogram in panel (a) marks the surface unit cell. (a) Filled (*eU* = −1 eV) and (b) empty state (*eU* = +1 eV) simulated STM topography (4 nm × 2 nm) of a Si NR on the Si(111) surface.

According to Table 1, the structural model with the lowest surface energy (5hex) is composed of pure hexagonal rings. The model is shown in Figure 2. The atomic structure of the NR is in reversed AB registry with respect to the substrate lattice (Figure 2b). This layer stacking has also been proposed as one of possible realizations of multilayer silicene [60]. The mean NR–substrate distance yields 2.89 Å, which suggests rather strong chemical bonding between these subsystems. The interaction with the substrate is reflected in the presence of Si atoms sticking out of the NR layer by 1.06 Å, as discussed for other systems [45,59]. The strong NR–substrate interaction also results in a substantial deformation of the outermost hexagons, which leads to a sawtooth shape of the NR edges. This arrangement of atoms increases the distance between atoms sticking out, *d*_BP_, which in the present case yields 8 Å. This value is by 0.3 Å longer than the expected double lattice constant in the *ZZ* direction, but still 0.6 Å less than the observed value. In reality the difference between experimental and theoretical values is expected to be smaller due to the scanner calibration, which is expected to overestimate distances up to 3%. Nevertheless, this value of *d*_BP_ is the closest to the experimental BP–BP distance among the models studied. Note that most of the models either feature a single BP in the unit cell or produce no BPs at all.

The 5hex model accounts for main experimental findings, i.e., it has 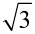 periodicity along the *AC* direction, produces two BPs per unit cell with a reasonable distance between them, and contains no Pb atoms. To further check the validity of the model we have performed STM simulations, which are presented in Figure 2d,e. Indeed, the calculations reproduce well the experimental data. In particular BPs, which reflect sticking out atoms, are well resolved. Similar as in the experiment, they form a rectangular lattice with 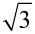 periodicity along NRs. However, in experimentally determined topography, the BPs across a NR are well separated, while calculations give additional features in between the BPs. These third protrusions come from Si atoms in the middle of NRs (compare Figure 2a,c). These atoms stick out of the NR layer by 0.6 Å, compared with 1.05 Å for BPs. Nevertheless, they contribute to the STM signal, in particular at positive sample bias, and make the topography more blurred. Another possibility for the discrepancy might be that interference processes suppress the STM current in the middle of a NR, an effect that cannot be captured by the Tersoff–Hamann approach.

So far we have discussed only pure Si structures, disregarding the role of Pb atoms in the system. We have only mentioned that Pb atoms may stabilize the NRs, because the NRs on a bare Si(111) surface increase the surface energy, and the pure Si system should be unstable. We have checked the stability of the 5hex model in the presence of Pb atoms. In this case the relative surface energy γ_NR(Si−Pb)_ reads

**Figure 3:**
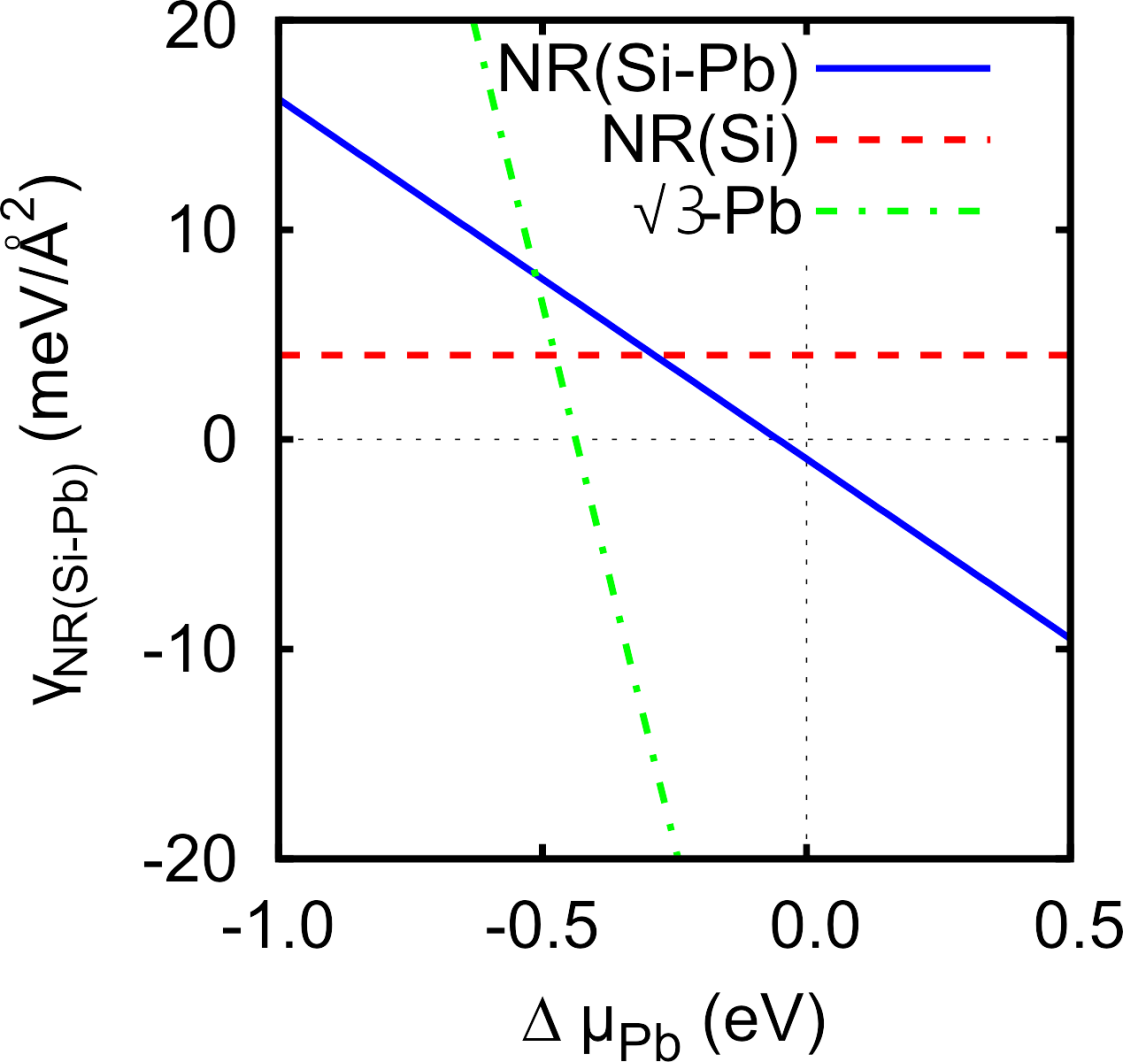
Relative surface energy γ_NR(Si−Pb)_ vs the chemical potential of Pb Δμ_Pb_ measured with respect to its bulk value 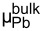.

[2]
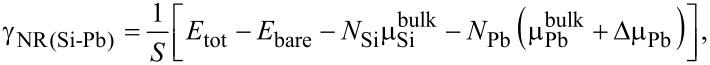


where *E*_tot_ is now the total energy of a NR and Pb atoms on a Si(111) surface, *N*_Pb_ denotes the number of Pb atoms in a unit cell, and Δμ_Pb_ is the chemical potential of Pb measured with respect to its bulk value 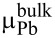. In this way calculated relative surface energy γ_NR(Si−Pb)_ is shown in Figure 3.

Clearly, as the chemical potential of Pb, Δμ_Pb_, increases and approaches its bulk value, the relative surface energy γ_NR(Si−Pb)_ becomes negative, indicating the stability of the system. Note that the most stable system should be the 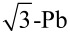 reconstruction, but this is in line with experimental findings suggesting that Si NRs growing on the bare surface move Pb atoms, which form the dense 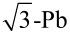 phase in between the NRs. The stability of Si NRs is achieved by passivation of the bare Si(111) surface by Pb atoms, which in turn lowers the surface energy. The main process behind the energy lowering is the charge transfer from Pb to Si atoms. According to the Bader analysis, the largest amounts of charge, 0.26*e* and 0.09*e*, are transferred to the NR-edge Si atoms and to the third protruding atom in the middle of a NR, respectively.

**Figure 4:**
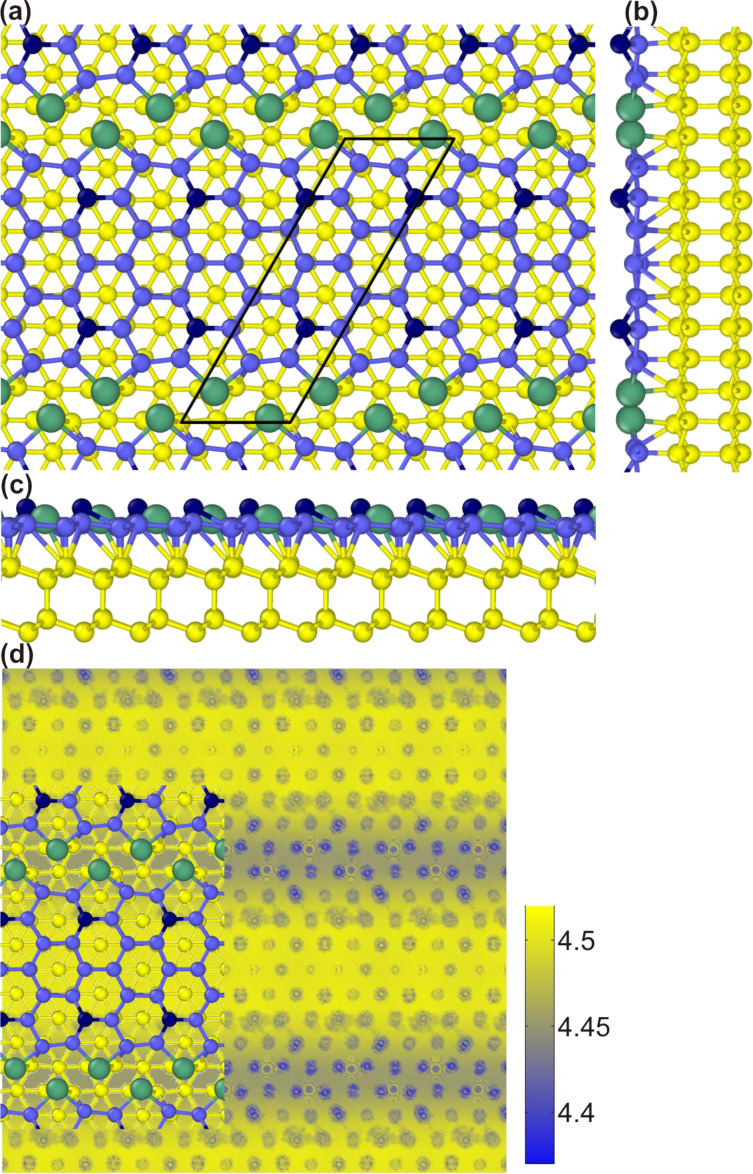
(a) Top and (b), (c) side views of the 5hex model in the presence of Pb atoms (shown in green). (d) Local distribution of the electrostatic potential in the vacuum region.

The main features of the 5hex model remain unchanged in the presence of Pb atoms, as Figure 4 shows. In particular, two Si atoms forming BPs and deformed outermost hexagons are still present. Moreover, the sticking out Si atoms do not change their positions with respect to the flat NR layer. They stick out by 1.05 Å, the same value as in the Pb-free case, and maintain their original separation *d*_BP_. However, looking into the details of the NR structure in the presence of Pb, it turns out that important modifications appear. The whole NR is pushed away from the surface, and the mean NR–surface separation increases by 0.1 Å with respect to its Pb-free value. This results in a weakening of the NR–substrate interaction. In fact, this should somehow be expected since Pb atoms passivate the Si(111) surface. This weaker interaction and the substantial charge doping lead to the depression of the third protrusion in the middle of a NR of the original 5hex model (compare Figure 2b and Figure 4b). Note that this protrusion had substantially spoiled the agreement between theoretical and experimental STM topography images. As a result of Pb passivation an STM topography with two protrusions is now obtained.

To further check the validity of the model, we have calculated the work function Φ for Si NRs and for Si–Pb NRs, which yielded 4.70 eV and 4.45 eV, respectively. Thus adding Pb atoms to areas between Si NRs decreases the value of Φ. We also calculated the local electrostatic potential distribution in the vacuum region (Figure 4), which can be compared to the d*I*/d*z* maps of Figure 1d. Clearly, Si NRs feature higher values of Φ then Pb areas, in full agreement with the experimental results.

**Figure 5:**
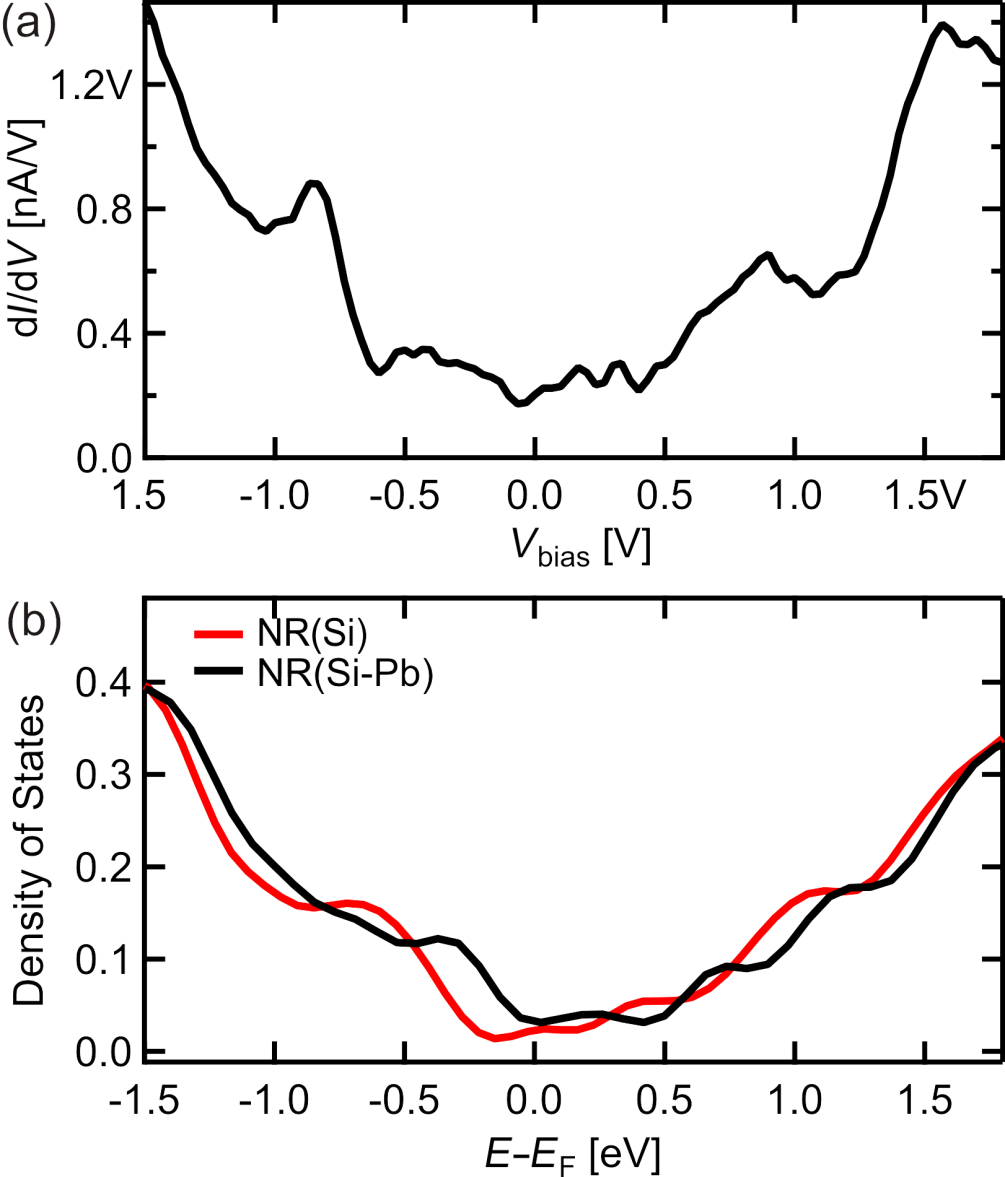
(a) d*I*/d*V* point spectroscopy data acquired on top of the Si NR. (b) Total density of states of Si NR system in the absence (red line) and in the presence of Pb atoms (black line). Note that the system is metallic.

To shed light on electronic properties, we provide a comparison of the measured d*I*/d*V* characteristics and calculated density of states (DOS) in Figure 5. Again, the theoretical results reproduce well the experimental data. The system is metallic with overall V-shape behavior and some fine structure imposed on it. Note that Pb atoms only slightly modify the DOS characteristics.

The above results show that Pb atoms play an important role in the formation of Si NRs. By passivating the substrate and donating charge they lower the surface energy, suppress the NR–surface interaction, stabilize Si NRs and modify their properties. In short, they improve the agreement between theoretical and experimental results. However, it is important to stress that such scenario could only be realized owing to the fast diffusion of Pb atoms on Si substrates. Simply, Pb atoms must make room for growing NRs directly on the substrate. If the diffusion was too slow, Si would grow on top of Pb, not necessarily in a 1D or 2D fashion. Thus both, thermodynamics and kinetics, play a significant role in the formation of Si NRs. We believe that a mechanism utilizing fast diffusion of atoms on other substrates may serve as an efficient way of growing silicene nanostructures.

## Conclusion

In conclusion, we have studied structural and electronic properties of silicene-like nanoribbons formed on a Si(111) surface with Pb-induced reconstrcution. Based on density functional theory calculations, we have proposed a structural model of the nanoribbons. The model features a deformed honeycomb lattice in the reversed AB registry with the top Si(111) layer, and the presence of Si atoms sticking out from the surface, which are visible as bright protrusions in the STM topography. The nanoribbons are directly bonded to the substrate, while Pb atoms stabilize the system by passivating the uncovered substrate and donating electrons to Si atoms. Thus, they lower the surface energy and suppress the nanoribbon–substrate interaction. The proposed model reproduces well all the experimental data. These findings provide a deeper insight into the formation of silicene nanostructures on metal-induced silicon surfaces and open new routes to grow silicene on other substrates utilizing the mechanism of fast atomic diffusion.
